# Health Access and Outcome Differentials Among Older Adults Age 50 Years or Above in Ghana

**DOI:** 10.1093/geroni/igaa057.351

**Published:** 2020-12-16

**Authors:** Kodwo-Nyameazea Yale, Nana Akua Amponsah

**Affiliations:** 1 University of North Carolina at Pembroke, Pembroke, North Carolina, United States; 2 Coastal Carolina Neuropsychiatric Center, Fayetteville, North Carolina, United States

## Abstract

Health outcomes are substantially influenced by demographic and socioeconomic indicators. Their presence or absence can act to boost or curtail health opportunities and outcomes. These health indicators are often not evenly distributed across geographic regions. The focus of this study is to explore how Ghana’s north-south regional divide manifests in the health outcomes of its people. The study was based on a data from the Research on Early Life and Aging Trends and Effects (RELATE), a harmonized cross‐sectional dataset with 147,278 respondents from 14 countries including Ghana. This study pooled 4724 respondents from Ghana aged 50 years or above. The study found that several demographic and socioeconomic indicators had significant associations with optimal health. However, most of the associations were attenuated after other variables in the model were controlled. Specifically, the study found that being a male gender and doing rigorous work were risk factors for living below optimal health in both the North and South regions. However, using healthcare services and being single or unmarried were protective factors but only in the South region. The results of the study also showed that indicators for good health generally favored the South region but the North region reported higher self-rated health score, smaller proportions of overweight and obese residents, fewer numbers of chronic health conditions, and a smaller proportion of resident living below optimal health. This finding demonstrates that the link between positive health indicators and healthiness has subtle nuances that could be attributed to how people conceptualize health and healthiness.

